# Study on the vertical bearing performances of piles on karst cave

**DOI:** 10.1038/s41598-023-31458-2

**Published:** 2023-03-27

**Authors:** Huiyun Chen, Zhongju Feng, Min Wu, Guimei Zhou, Lu Chen, Cong Zhang

**Affiliations:** 1 School of Architecture and Civil Engineering, Xihua University, Chengdu, 610039 China; 2 School of Highway, Chang’an University, Xi’an, 710064 China; 3 The Limited Company of Institute Architectural Design and Research of Shanxi Province, Taiyuan, 030000 China; 4 Fujian Provincial Transportation Planning and Design Institute Co. LTD, Fuzhou, 350000 China

**Keywords:** Civil engineering, Solid Earth sciences

## Abstract

Caves affected the load transfer mechanism of bridge pile foundation, and then the safety of the bridge was threatened. This study was to investigate the effect of karst cave under bridge pile foundations on the vertical bearing characteristics of bridge pile foundations by static load test, finite element analysis and mechanical model. The settlement of the pile was measured by displacement meter, and the axial force were measured by stress gauges in the test. The load-settlement, the axial force, the unit skin friction and the ratios of side and tip resistances were compared with the result of the simulation. Then sixteen conditions were selected in finite element analysis, one of them was a conventional pile not on cave. The others were about five kinds of height, five kinds of span and six kinds roof’s thickness of the cave. The simply supported and fixed wide beam were established to calculate the allowance roof thickness. The results reveal that when the cave span is greater than 9 m × 9 m or the roof thickness is less than 2 *D* (pile diameter), the stress and deformation of piles are significantly affected.

## Introduction

Karst is a complex substrate, and with the stable supports required for bridge structures, the performance of bridge pile foundations in karst is vitally important. Three problem scenarios can occur when installing bridge pile foundations in terrain with karst caves: cave across pile, cave at side of pile, or cave under pile. The bearing characteristics of piles are significantly different with the different size and roof of the underlying cave^1^. The cave under pile causes the lack of bearing layer at the pile bottom, which poses a threat to piles^2–5^.

The bearing performance of bridge pile foundations in karst area has attracted much attention of scholars. Feng, Chen and He successfully predicted the bearing strength and stability of piles that constructed on karst cave based on the high accuracy grey model^6–9^. Wong and Dong implemented the load-settlement law and calculation method of bearing capacity and safe thickness in karst areas using a static load test and theoretical deduction^10,11^. Chen and Hu studied the vertical destroy mode of partially-screwed pile and calculated its bearing capacity by test and simulation^12,13^. The influence of the thickness and size of the cave roof on the bearing capacity and the sensitivity of each factor were found by Zhang^14,15^. Liu and Fattah investigated the vertical performance of the pile with the changing size, roof thickness of and shape of the karst cave and analyzed the pile’s stability^16,17^.

Most scholars studied the failure mode and the stability of karst cave’s roof. Zhao and Xiao proposed the stabilities of the one void and multiple voids under different undrained condition and discussed the influence of inclined load on the void^18,19^. Jiang, Zhao, Zhang, Wang and Bai established the simply supported mechanical model to get the theoretical calculation formula of the safe thickness of cave roof and studied the destroy mode of the roof, when the roof is subjected to punching, shear and bending and tensile failure^20–24^. According to Wilson, Tschuchnigg and Rodrigo, the stability of a structure could be determined by limit equilibrium analysis in engineering^25–27^. Lee proposed the stability of the void under different undrained condition and discussed the influence of inclined load on the void^28,29^. Almost all of above researches are studied by the finite element analysis and mechanical model. Static load test is one of the most reliable methods to study the performances of piles^30–34^.

In this study, the effects of the height, span and roof thickness of the underlying karst cave on the vertical bearing characteristics of bridge pile foundations were investigated by the static load test and finite element analysis. The vertical bearing capacity were obtained under different caves. Meanwhile, the load transfer characteristics were studied under different scenarios including the axial force, unit shaft resistance and ratios of two resistances.

## Materials and methods

### Static load test

#### Engineering overview and geological conditions

The design of the field test is completely dependent on the prototype pile, so the data of the field test are the most reliable and representative. In order to reflect the bearing characteristics of the pile foundation under the influence of the underlying cave truly and verify the accuracy of the finite element simulation model, the field static load test on the bearing characteristics of the pile foundation were carried out. The static load tests in this manuscript innovatively used of the internal benchmark beam^35,36^. It enables more efficient transfer of upper loads to the test piles. Both the displacement meter, the strain gauge and earth pressure cells were used in this test, so that the mechanics and deformation data of the pile foundation obtained by the test were relatively comprehensive. The static load test mainly relied on a 470 m long flyover bridge in Pingdingshan. The superstructure of the bridge adopted cast-in-place continuous box girder, the lower part adopted column pier, and the foundation was the bored pile. The bridge was located in a flat with a ground elevation of about 89.30 m. The upper layer of the bridge site was quaternary and the lower layer was Cambrian dolomitic limestone. The characteristics of geological conditions are shown in Table 1. The bridge spanned the karst area, and there were many hidden karst caves. The caves were mainly distributed in piers 1#–16#. According to geological prospecting data and site construction, pier 3# was selected as the test pile. The characteristics of karst in the location of pier 3# are shown in Table 2.

**Table 1:** Geological conditions.

Name	Characteristics	Elevation/m	Thickness/m
Miscellaneous fill	Brown yellow, containing massive soil, slightly loose, widespread distribution	88.5–85.8	0.8–3.5
Silty clay	Brownish red and brownish yellow, with a small amount of gravel, 20–30% grayish white calcareous nodules, generally 20–30 mm in diameter, local gravel and breccia at the bottom, calcium core content can reach 50–60%, widespread distribution	68.0–75.3	10.5–20.5
Karstified dolomitic limestone	It is mainly composed of dolomite and calcite, partially containing breccia and boulder. The rock mass is broken and has a honeycomb structure. The void is filled with clay, and the dissolution phenomenon is obvious	61.2–73.7	1.6–6.8
Medium weathered limestone	Bluish gray, mainly dolomite, the rock is hard, the joints and cracks are developed, for cryptocrystalline structure. In the middle, a small number of rock fissures are developed. The karst are formed locally, which are mostly hollow or filled with clay	–	–

**Table 2:** Karst characteristics of pier 3#.

Number	The elevation of bedrock roof /m	The degree of relief of bedrock roof /m	The number of karst cave	The height of the cave /m	The lowest elevation of cave floor /m
3#	66.5–68.8	2.3	15	0.2–15.3	34.5

#### Physical properties of rock and soil

**Figure 1:**
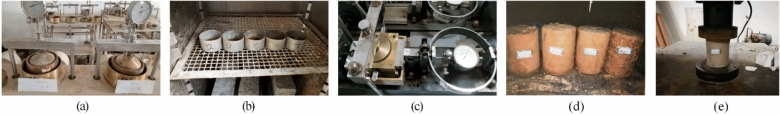
Physical property tests: (**a**) oedometer test; (**b**) moisture content test; (**c**) direct shear test; (**d**) specimens with different proportions; (**e**) compression tests.

The quaternary soil covered all the rock strata and concealed karst was developed at the location of SZ5 of pier 3#. The geological conditions were determined by drilling sampling method. As Fig. 1, the oedometer test could test out the compression modulus. The drying test could get the moisture content. The direct shear test could receive the shear parameters. The compression tests could test out the parameters of the rock. The parameters of soil and rock are shown in Tables 3 and 4. The bearing layer of SZ5 was moderately weathered limestone with a thickness of 1.5 m. Where, *E*_s_ is the Young's modulus; *t* is the thickness; *μ* is the poisson ratio; *c* is the cohesion; *φ* is the internal friction angle; *γ* is the unit weight; *f*_rk_ is the uniaxial ultimate compressive strength of the rock; *R*_t_ is the ultimate tensile strength of the rock.

**Table 3:** Parameters of soil.

Soil	*E*_s_/KPa	*t*/m	*μ*	*c*/KPa	*φ*/°	*γ*/kN·m^−3^
Miscellaneous fill	1.3 × 10^4^	3	0.4	10	8	18.5
Silty clay	4 × 10^4^	12.0	0.3	18	10	19.5
Karstified dolomitic limestone	8 × 10^6^	3	0.26	0	28	24.5

**Table 4:** Parameters of rock.

Rock	*E*_s_/KPa	*μ*	*f*_rk_/MPa	*R*_t_/MPa	*γ*/kN·m^−3^
Medium weathered limestone	2.2 × 10^7^	0.23	33	5.5	25

#### Test pile (SZ5)

SZ5 is a model bridge pile foundation with 19.5 m in length and 1.5 m in diameter. It is a cast-in-place pile with the ultimate load of 8120 kN in design. SZ5 is built on a karst cave with a height of 3.2 m and a span of 6.0 m × 3.0 m, and the bottom of the pile is 1.5 m to the top of the cave. The heights of the caves are measured in the axial direction of the pile. The spans of the caves are measured in two directions perpendicular to the axial direction of the pile. The roof is the rock from the pile bottom to the cave. The parameters of SZ5 is shown in Table 5. In Table 5, *l* is the length of SZ5; *D* is the diagram of SZ5; *Q*_u_ is the ultimate load of SZ5 in design; *h*_r_ is the rock-socketed depth of the pile; *H*_c_ is the roof thickness of the cave; *H* is the height of the cave; *B* is the span of transverse of the cave; *L* is the longitudinal length span of the cave.

**Table 5:** Parameter of SZ5.

Pile	Concrete grade	*l*/m	*D*/m	*Q*_u_/kN	*h*_r_ /m	*h*_c_/m	*H*/m	*B* × *L*/m^2^
SZ5	C25	20.0	1.5	8120	2.0	1.5	3.2	6 × 3

#### Test equipment

**Figure 2:**
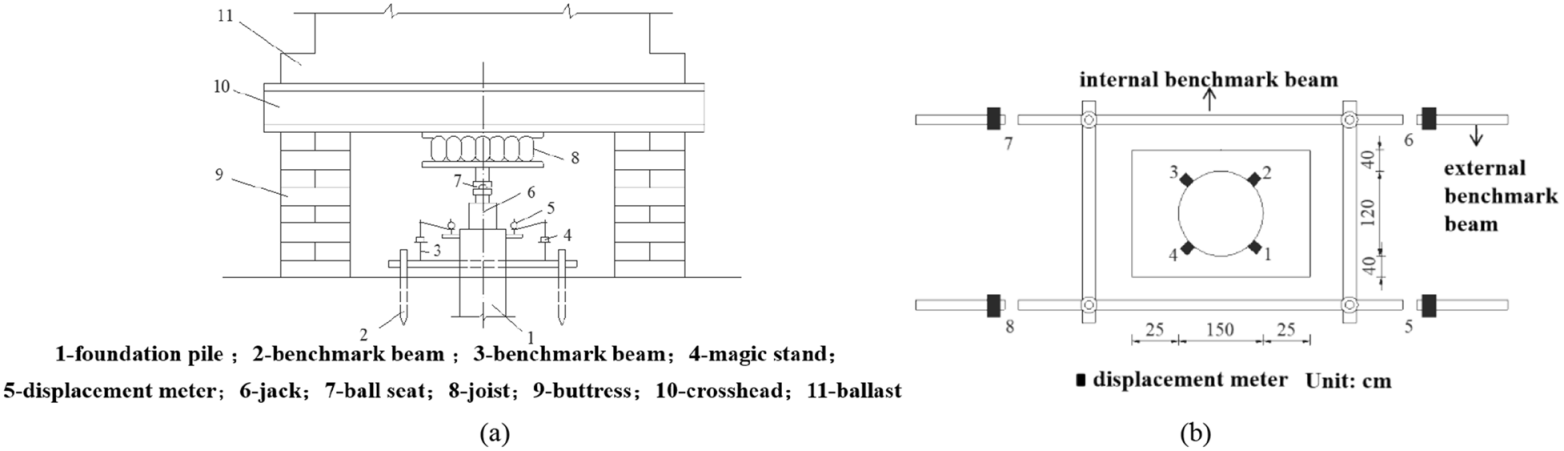
Test Equipment: (**a**) pile test platform and reaction device; (**b**) base beam layout.

Loading hydraulic system consists of 4 jacks (Fig. 2a6) with pressure stabilizing devices. The hydraulic gauge was in parallel with the jacks to determine the oil pressure and control the amount of loading. Cross-girder (Fig. 2a10) was made of rebar steel mounted on the stack platform. The concrete blocks were used as the ballast (Fig. 2a11) on the cross-girder platform. The base beam was made of steel, which includes the internal and external reference beam, as shown in Fig. 2b. The external reference beam set in the test has no settlement under the load. It could ensure the accuracy of the test results if the internal reference beam moves down under the load. Figure 2 shows the reaction device of the stack platform. Where *l* is length of SZ5; *D* is diameter of SZ5; *Q*_u_ is ultimate vertical bearing capacity of SZ5 in design; *M*_u_ is the bending moment of SZ5 in design; *h*_r_ is the depth of the embedded rock of SZ5; *h* is the thickness of the roof; *h*_c_ is karst cave’s height; *B* is longitudinal span of cave; *L* is the horizontal span of cave.

**Figure 3:**
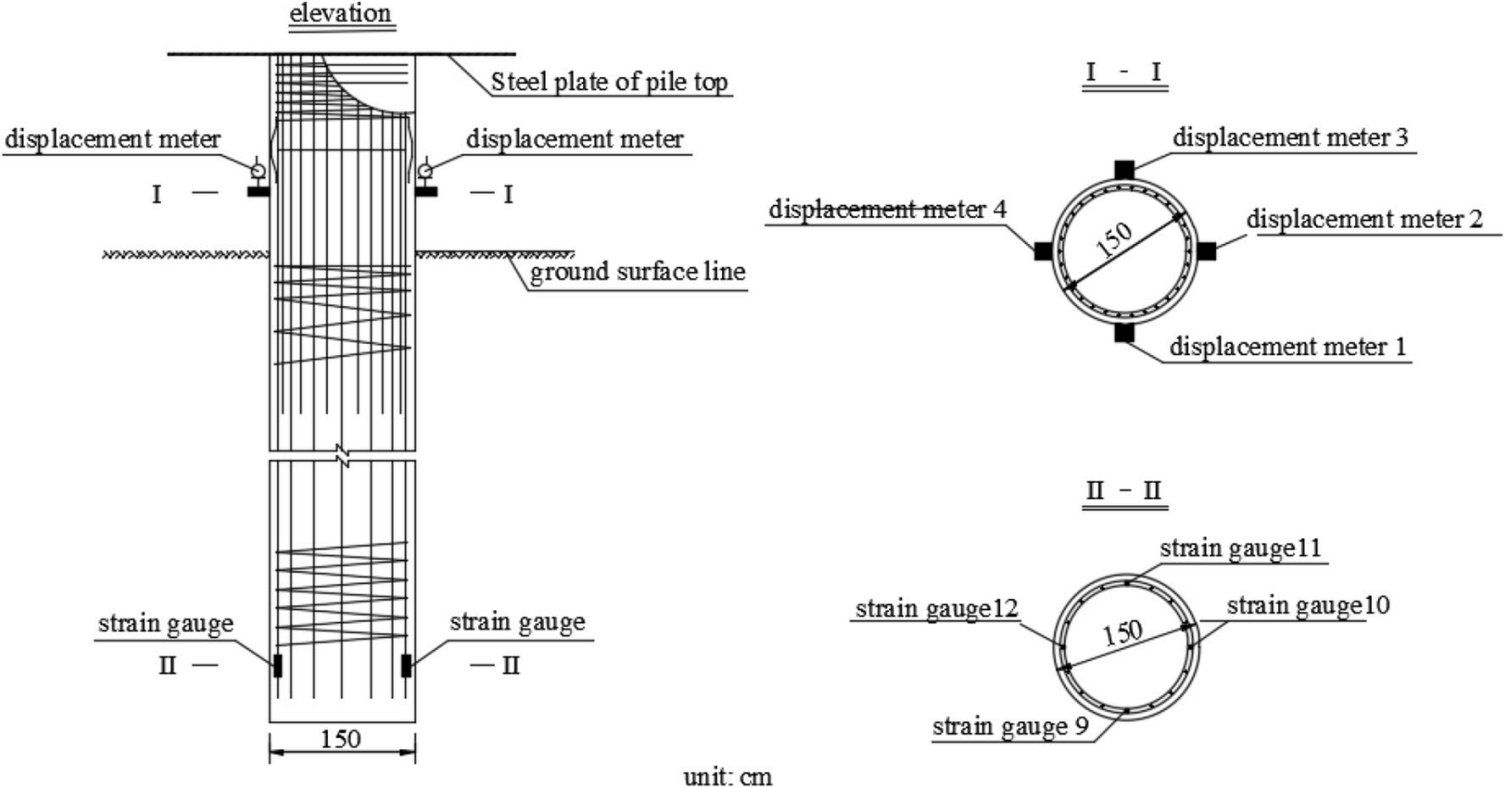
Displacement meter and strain gauge layout.

Four displacement meters were installed on SZ5, and four on the outer beam, as shown in Figs. 2 and 3. The displacement meters were arranged symmetrically and evenly. Strain gauges laid on the top and side of SZ5 near the bottom, as shown in Fig. 3.

**Figure 4:**
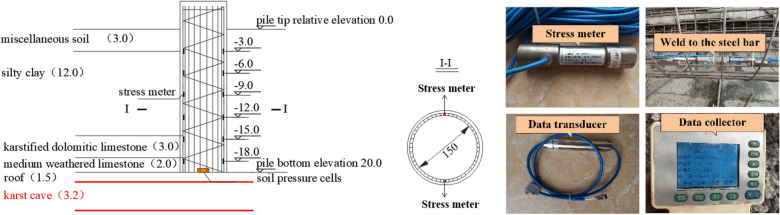
Arrangement of stress gauges and earth pressure cell.

Stress meters were arranged along the two symmetrical main reinforcements of SZ5 for the accuracy of the data. The stress meters were welded to the steel bar as shown in Fig. 4. The force of reinforcement could be required from the data collector. Two stress meters were symmetrically installed at 3 m intervals. The earth pressure cell was set in the center of the tip of SZ5.

#### Test load

The test was carried out by slow maintenance loading method. The vertical load included 11 levels. Each level increased by 2 MN. The settlement of SZ5 was recorded at 5 min, 15 min, 30 min, 45 min and 60 min in the first hour, and then recorded once every 30 min. When the settlement was less than 0.1 mm at least twice within an hour, it could be regarded as stable. Then the load was removed at 2MN per level. After removing each level load, the residual settlement was required at the 15 min, 30 min, 60 min. After the load was decreased to zero, the residual settlement was read every 15 min in the first half an hour, and then once every 30 min, which took 3 h altogether.

### Finite element limit analysis

#### Model design

**Figure 5:**
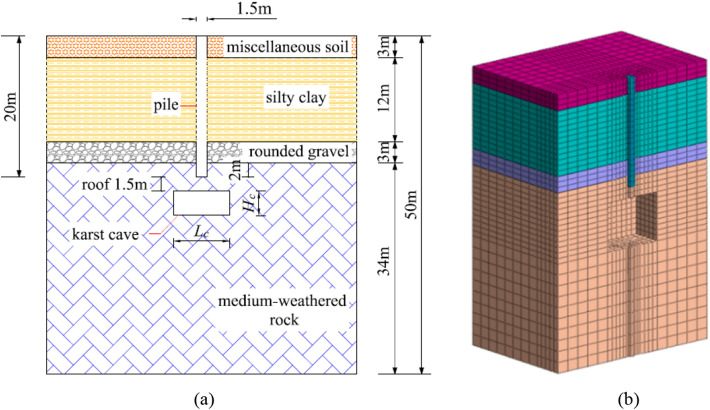
Model pile and soil distribution: (**a**) soil distribution and position of pile in finite element analysis; (**b**) model.

The pile was made of concrete as a solid model, which adopt the ideal elastic constitutive relation. The elastoplastic constitutive model and Mohr–Coulomb yield criterion were selected for the rock and soil. The soil and rock were established as 2D grid by ABAQUS, and extended to 3D entity unit in order to keep the contact surface continuous. Then each entity was added with a corresponding material attribute (Fig. 5, Tables 1, 2, 3). The unit extension of the pile was continuous. The 2D element at the location of the cave in the holdup rock was hidden when making the cave, and there was no material attribute in the cave. The cave was simplified to a quadrilateral prism to simplify the calculation. The master–slave contact algorithm was used for the interface between pile and soil.

#### Three factors in finite element analysis

The parameters that are manipulated included the height, the span, and the roof thickness of karst caves that interact with piles. There are seventeen total treatments, including one with piles without karst caves (control), five different heights (3, 6, 9, 12, 15 m), five different spans (3 × 3, 6 × 6, 9 × 9, 12 × 12, 15 × 15 m × m), and six different thickness of caves (0.75 m, 1.5 m, 2.25 m, 3.0 m, 3.75 m, 4.5 m). The pile is 20 m in length and 1.5 m in diagram. It is embedded in rock 2.0 m. When the span of cave is manipulated, the height and roof thickness of cave is 3 m and 2.25 m, respectively. When the height is changed, the span and roof thickness of cave is 6 m × 6 m and 2.25 m, respectively. The heights of the caves are measured in the axial direction of the pile. The spans of the caves are measured in two directions perpendicular to the axial direction of the pile.

## Results and discussion

### Test results

#### Load—settlement law

There are two methods requiring the ultimate vertical bearing force of piles from load-settlement curve according to the standard. One of which is the force corresponding to the sudden change settlement. The other method is to calculate the value of 3% of the pile diameter and the value of 40 mm, then elect the smaller one to regard as the failure settlement^37^ The load needed to produce this settlement is the bearing capacity of piles when the settlement does not mutate. When the settlement is more than two times of the adjacent settlement, the pile foundation has been damaged. The adjacent load is the ultimate bearing capacity of the pile.

**Figure 6:**
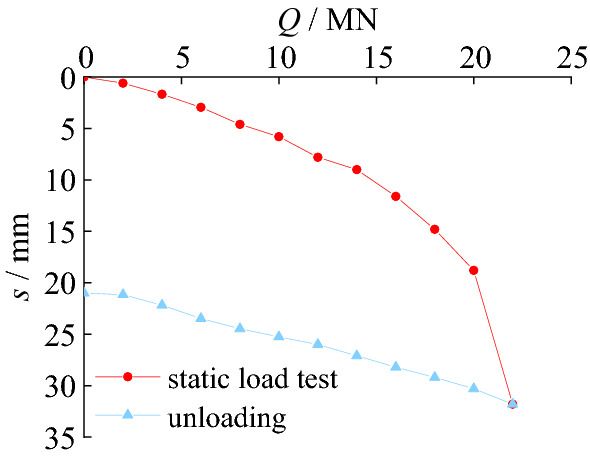
Load-settlement curves of test pile.

The *Q-s* curves in static load test can be seen in Fig. 6.

As shown in Fig. 6, the final loading in the test is 22 MN, and the corresponding settlement of SZ5 is 31.8 mm. The residual settlement after unloading is 21.0 mm. The vertical ultimate bearing capacity of SZ5 is 20.35 MN. The main reason is that the settlement of SZ5 is mainly elastic deformation of the pile with small load. With the increase of load, more load is transferred to the bottom of the pile.

#### Load transfer law

The axial force and unit pile side friction of SZ5 can be obtained by Eq. (1) and Eq. (2) ^8,38^.

1 $$Q_{i} = n \times F_{G} + (E_{H} \times A_{H} \times F_{G} )/(E_{G} \times A_{G} )$$

2 $$T_{i} = (Q_{i} - Q_{i + 1} )/Ul_{i}$$

where *Q*_*i*_ is the axial force of pile; *F*_G_, *E*_H_ and* A*_H_ is axial pressure, the elastic modulus and the cross-sectional area of a main reinforcement, respectively (*E*_H_ is 2.8 × 10^4^ MPa and the diameter of the main bar is 28 mm). *E*_G_ and *A*_G_ is the elastic modulus and the cross-sectional area of concrete, respectively (*E*_G_ is 2.0 × 10^5^ MPa); *n* is the reinforcement number (*n* is 28). *T*_*i*_ is the unit side friction (*i* is the number of stress meter, 1 to 6 from pile top to tip), it is the force on the unit side area of the pile element; U is the circumference of the pile; *l*_i_ is the distance between adjacent stress meter (Fig. 4).

**Figure 7:**
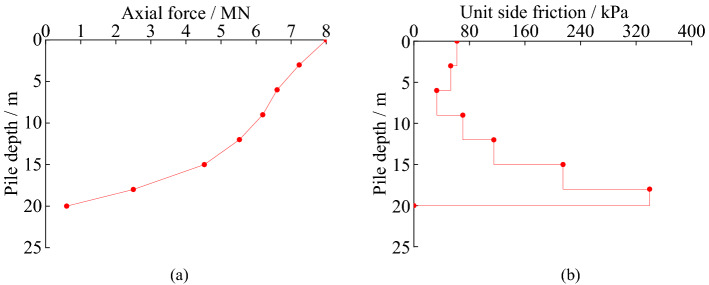
Load transfer law of test pile: (**a**) axial force of piles; (**b**) unit side friction of piles.

The axial force and unit side friction of SZ5 in static load test can be seen in Fig. 7.

In Fig. 7, the axial forces of the piles decrease gradually along the pile. The attenuation rate of the axial force increases significantly in the range of rock strata. When the load is 8.12 MN, the side friction of SZ5 accounts for 92.5%. The main reason is that the relative displacement between pile and soil decreases with the increase of pile depth. The parameters taken in the design are conservative. When the load is 8.12 MN (ultimate load of SZ5 in design), SZ5 shows typical characteristics of friction pile.

### Analysis of bearing capacity of piles under three factors of underlying caves

**Figure 8:**
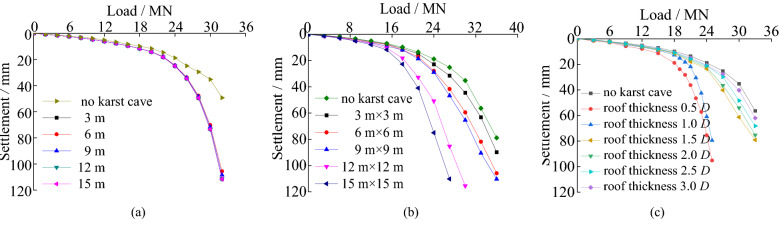
The load–settlement curves under three factors: (**a**) different cave’s height; (**b**) different cave’s span; (**c**) different cave’s roof thickness.

**Figure 9:**
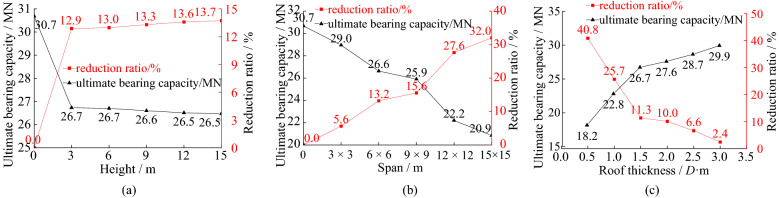
The ultimate bearing capacity and reduction ratio under three factors: (**a**) different cave’s height; (**b**) different cave’s span; (**c**) different cave’s roof thickness.

The load–settlement curves under three factors and the ultimate bearing capacity and reduction ratio under three factors can be seen in Figs. 8 and 9, respectively.

As shown in Fig. 8, the load-settlement curves have no sudden change point with the changing of cave’s height and span except the roof thickness smaller than 1.5 m. In Fig. 9, the underlying caves lead to greater settlement at the top of pile compared with the pile without cave under the same load and the ultimate vertical bearing capacity decreases a lot. The ultimate bearing forces show a tiny decreasing trend with increasing cave height. It decreases only 0.8% when the height increases from 3 to 15 m. The main reason is that the increasing cave height has little influence on the roof’s strength. Even if the height of the underlying cave increases, the plastic development zone remains almost unchanged.

Under identical loads, bigger karst caves spans result in greater pile settlement. The ultimate vertical bearing forces of piles reduce significantly with increasing cave span. It decreases 3.1 MN when the span increases from 3 m × 3 m to 9 m × 9 m. The reduction ratio of the ultimate vertical bearing forces reaches up to 16.4% when the span increases from 9 m × 9 m to 15 m × 15 m. The ultimate bearing capacity of piles decreases faster after cave’s span exceeding 9 m × 9 m. The main reason is that the increasing span leads to the decrease of the thickness-span ratio of the cave. The roof becomes a thin plate when the cave’s span exceeding 9 m × 9 m, and it is more prone to bending-tensile failure.

As the roof thicknesses increase, the settlements of the piles decrease under identical loads. The ultimate vertical bearing capacities of piles increase significantly with increasing roof thickness. It increases 8.5 MN with increasing roof thickness from 0.75 m to 2.25 m. But it is only 3.2 MN, when the roof thickness increases from 2.25 m to 4.5 m. The reduction ratio of the bearing forces is less than 10%, when the thickness exceeds 3.0 m. It is because the increasing roof thickness enhances the roof’s strength. There is smaller settlement of pile with thicker roof.

### Analysis of load transfer law under the three factors

#### The axial force curves and unit side friction under different cave height

**Figure 10:**
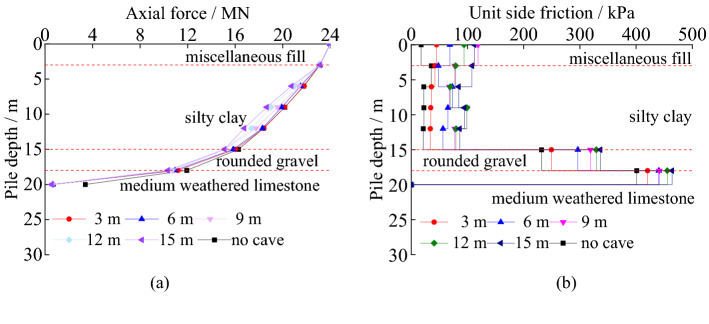
The load transfer characteristics of piles under different cave height: (**a**) axial force; (**b**) unit side friction.

The load transfer characteristics of piles under different cave height can be seen in Fig. 10. The axial force has slowly decreased in the pile’s length orientation in overburden, and it decreases faster when the pile was in rounded gravel and medium weathered limestone. The axial force of the pile on a cave decreases more than there is no cave under the pile. With the increasing cave height, the axial force decreasing velocity increases slightly. The taller the karst cave is, the smaller the axial force that is transferred to the pile bottom. The unit side friction of pile generally decreases first and then increases, and it reaches the peak value in the medium weathered limestone. Compared with non-karst under the pile, the unit side friction of pile on a cave is greater in the same depth. The unit friction is more fully exerted with the increasing cave height. The main reason is that the increasing cave height has little effect on the roof of the holding force.

#### The axial force curves and unit side friction under different cave span

**Figure 11:**
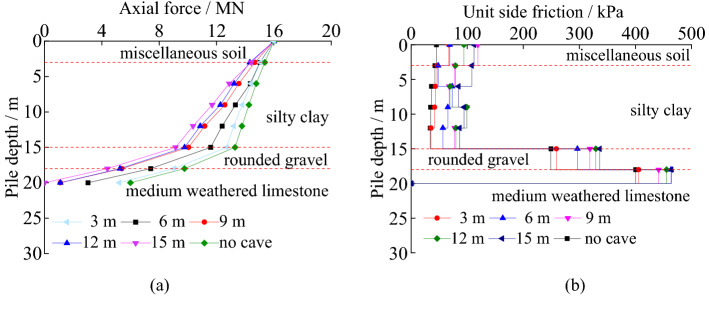
The load transfer characteristics of piles under different cave span: (**a**) axial force; (**b**) unit side friction.

The load transfer characteristics of piles under different cave span can be seen in Fig. 11.

Figure 11 shows the load transfer laws of piles with different underlying cave spans. The axial force of the pile decreases along the pile. The larger the cave span is, the smaller the axial force is transferred to the pile bottom. When the cave span exceeds 9 m × 9 m, the decrease of the axial force is accelerated obviously. The unit friction of pile is greater with the increasing cave span. The main reason is that the increasing span leads to the underlying roof’s deflection larger, the displacement between pile and soil increases. The unit side friction of pile is more fully developed. When the span is larger than 9 m × 9 m and the load is 16 MN, the cave roof bends more obvious.

#### The axial force curves and unit side friction under different roof thickness

**Figure 12:**
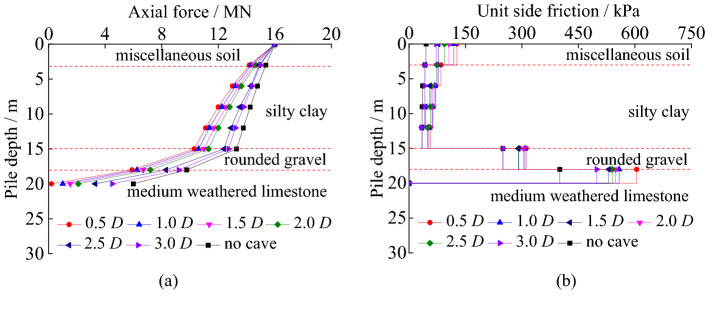
The load transfer characteristics of piles with different roof thickness: (**a**) axial force; (**b**) unit side friction.

The load transfer characteristics of piles with different roof thickness can be seen in Fig. 12.

In Fig. 12, the trends of the axial force of piles in soil and rock are similar with different roof thickness of karst caves. The difference is that with the increased roof thickness, the abrupt transfers in axial force towards the pile bottom obviously increase. The thicker the roof is, the greater the axial force at the same depth is. When the roof thickness exceeds 3.0 m, the axial force of the pile is reduced slower. With the increase of pile depth, the unit side friction of pile generally decreases first and then increases, and it reaches the peak value in the medium weathered limestone. The unit friction of pile is greater with the decreasing roof thickness. It is because that the roof of cave becomes a thin plate with the decrease of the roof thickness.

### Analysis of tip resistance and shaft resistance under ultimate bearing capacity of piles

The tip resistance of the piles under the ultimate vertical load was obtained by fitting through interpolation. The side resistance could be obtained by Eq. (3). The ratios were calculated Eq. (4) and (5).

3 $$Q_{si} = Q_{ui} - Q_{ti}$$

where, *Q*_s*i*_ is the side resistance and it is the lateral force on the pile; *Q*_*ui*_ is the ultimate bearing force; and *Q*_*ti*_ is the tip resistance; *i* is the condition number.

4 $$\alpha_{i} = Q_{si} /Q_{ui} \times 100\%$$

5 $$\beta_{i} = Q_{ti} /Q_{ui} \times 100\%$$

where $\alpha_{i}$ is the ratio of side resistance; and $\beta_{i}$ is the ratio of tip resistance.

**Figure 13:**
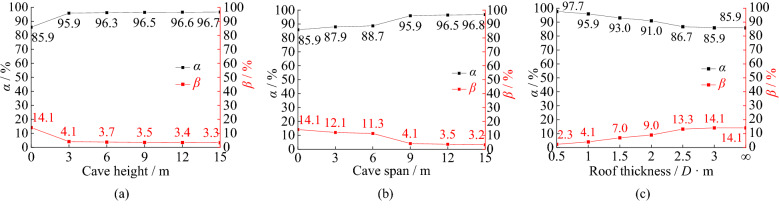
Ratios of tip resistance and side resistance of piles: (**a**) different cave height; (**b**) different cave span; (**c**) different roof thickness.

As shown in Fig. 13a, with increased karst cave height, the side resistance ratio of the pile shows a tiny increasing trend and the tip resistance ratio of the pile shows a tiny decreasing trend. The side resistance ratio increases 10.0% with the cave height increasing from 0 to 3 m. Then the tip resistance ratio reduces 1.4% with the increasing cave height from 3 to 6 m, but it only changes 0.3% with the increasing cave height from 6 to 15 m. Because the bearing stratum is likely a beam or board with the cave under the pile, the bearing capacity of the stratum are weakened. The increasing underlying cave height has little influence on the roof’s bearing capacity.

In Fig. 13b, the side resistance ratio increases 8.0% with the cave span increasing from 3 m × 3 m to 9 m × 9 m. The tip resistance ratio reduces 0.9% with the increasing cave span from 9 m × 9 m to 15 m × 15 m. When the span of karst cave exceeds 9 m × 9 m, the two proportions are to be stable. The main reason is that the roof of the cave can be regarded as a thin plate damaged more easily with the increasing cave span. The friction between the pile and the soil is fully exerted because of the bigger relative displacement between pile and soil. The side friction resistance shares most of the pile top load.

Figure 13c shows the side resistance ratio of the pile shows a decreasing trend and the tip resistance ratio of the pile shows an increasing trend with the increased roof thickness. When the roof thickness exceeds 3.0 m, the proportions is close to that of the non-karst pile foundation. The main reason is that the thicker the roof is, the greater the load required for plastic zone penetration is. The roof is penetrated by the pile with the thickness of 0.75 m and 1.5 m under the vertical ultimate load. The side friction exerts more fully with the roof thickness decreasing.

### The failure of cave roof under the ultimate load

**Figure 14:**
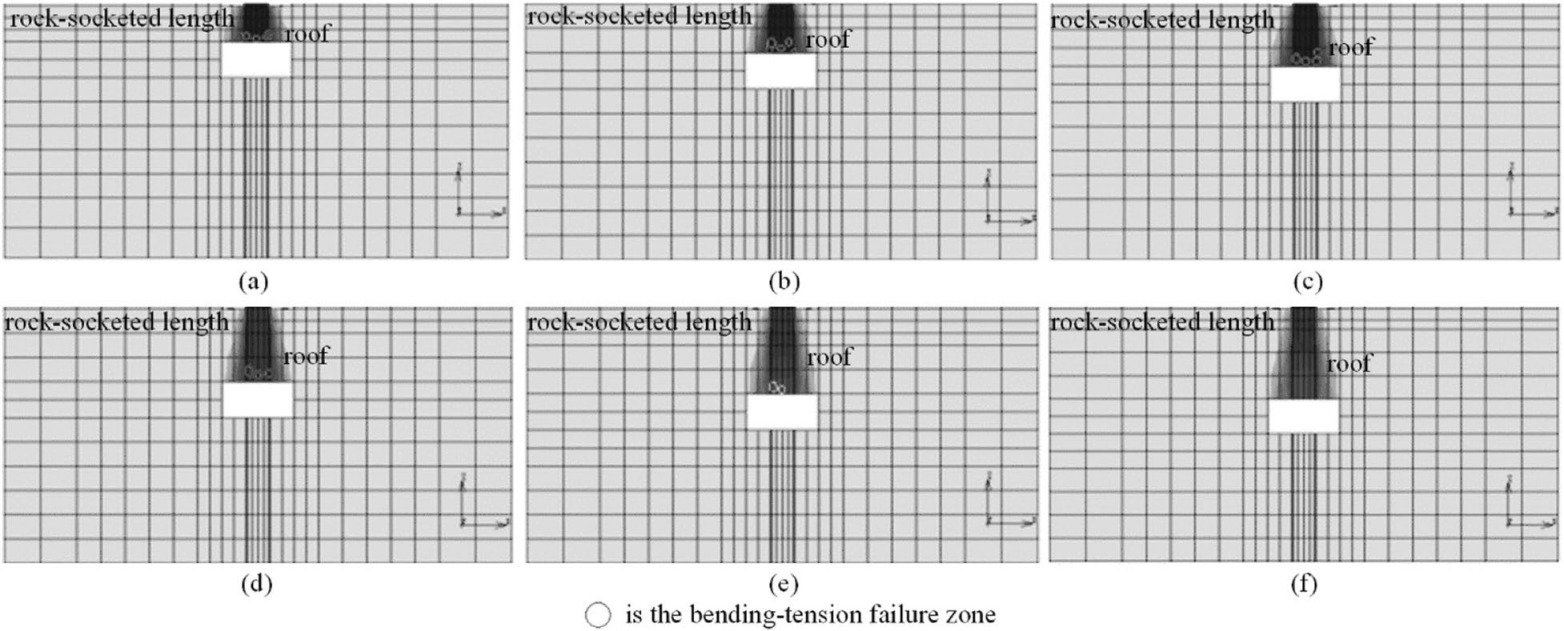
The failure of cave roof: (**a**) roof thickness = 0.75 m; (**b**) roof thickness = 1.5 m; (**c**) roof thickness = 2.25 m; (**d**) roof thickness = 3.0 m; (**e**) roof thickness = 3.75 m; (**f**) roof thickness = 4.5 m.

The failure situations of roof with different thickness under the vertical ultimate bearing capacity of the piles are shown in Fig. 14. The load transferred to pile tip leads to the bending and tensile failure zone of cave roof under the vertical ultimate load of the piles. The bending and tensile failure zone of cave roof mainly appears in the middle and bottom of the roof. As the thickness of cave roof increases, the ratio of pile tip resistance increases, but the bending and tensile failure zone of cave roof decreases. The main reason is that the load transferred to the pile tip concentrates on the middle part of the cave roof. Bending occurs in the middle of the roof. Because the tensile strength of the rock stratum is smaller than the compressive and shear strength, the tensile failure is easy to occur in the middle and bottom of the cave roof. The ultimate bearing capacity of the roof increases with the thickness of cave roof increasing.

### Comparative of results of the test and finite element analysis

#### Load—settlement law

**Figure 15:**
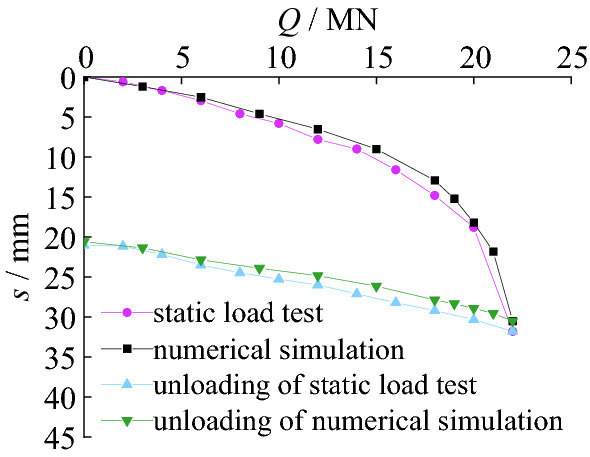
Comparison of load-settlement curves.

Figure 15 shows load-settlement curves of SZ5 and simulation pile. The relationship between the load and settlement of the pile foundation under the action of graded loads in field test and the finite element simulation both shows a similar trend. The *Q-s* curve of pile in the finite element analysis is almost coincident with it in the static load test. The settlement of the pile foundation in field test is 31.8 mm, and that of pile foundation in finite element simulation is 30.5 mm under 22MN load. The difference between them is 4.1%. The residual settlement of the pile foundation after unloading in static load test is 21.00 mm, and that of the pile foundation after unloading in finite element simulation is 20.58 mm. The difference between the two is 2.0%. The vertical ultimate bearing capacity of SZ5 is 20.35 MN of the test pile and 21.00 MN of the pile in the finite element analysis, respectively. The difference between the two results is only 3.2%.

#### Load transfer law

**Figure 16:**
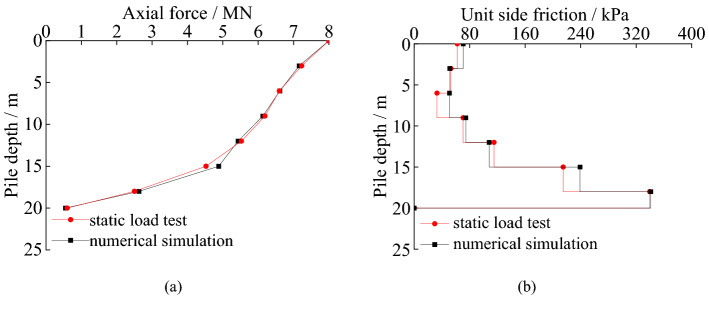
Load Transfer Law: (**a**) Comparison of axial force of piles; (**b**) Comparison of unit side friction of piles.

Figure 16 shows the axial force and unit side friction of SZ5 and simulation pile. The *Q*-*s* curve of pile in the finite element analysis is almost coincident with it in the static load test. The settlement of the pile foundation in field test is 31.8 mm, and that of pile foundation in finite element simulation is 30.5 mm under 22MN load. The difference between them is 4.1%. The residual settlement of the pile foundation after unloading in static load test is 21.00 mm, and that of the pile foundation after unloading in finite element simulation is 20.58 mm. The difference between the two is 2.0%. The vertical ultimate bearing capacity of SZ5 is 20.35 MN of the test pile and 21.00 MN of the pile in the finite element analysis, respectively. The difference between the two results is only 3.2%. Lines 279–280 of the revised manuscript. We have explained the analysis of the *Q*-*s* curve of results of testing with load cells and verification of the calculated models of the pile over the karst cavity in Lines 275–282 of the revised manuscript.

The axial force of SZ5 decreases by 3.475MN from the pile top to the depth of 15 m, and the reduction ratio is 43.3%. The load transferred to pile bottom is 0.60MN in the testing. The axial force of the simulation pile decreases by 3.117MN from the pile top to the depth of 15 m, and the reduction ratio is 39.0% of the. The load transferred to pile bottom is 0.55MN in the finite element simulation. The law of the axial force of the simulation pile is consistent with that in the field test. The inflection point of the axial force of the pile is both located at the position where the pile foundation enters the rock. The unit side friction of the pile is small in the overlying soil and becomes bigger when the pile socked in the rock. The distribution law of unit side friction of the pile is similar between the SZ5 and the simulated pile. When the load is 8.12 MN, the side frictions of SZ5 and finite element analysis pile account for 92.5% and 93.6%, respectively. The main reason is that the relative displacement between pile and soil decreases with the increase of pile depth. The parameters taken in the design are conservative. When the load is 8.12 MN (ultimate load of SZ5 in design), SZ5 shows typical characteristics of friction pile. The finite element analysis results are in good agreement with the static load test results.

## Conclusions

In this study, the effects of the underlying cave’s height, span, and roof thickness on the bearing characteristics of piles were investigated by load tests and finite element modelling. The conclusions of this study are as follows:

1. The settlement of the SZ5 in the static load test sudden increases under the load of 22 MN. It indicates that the cave roof is destroyed. Ensuring the allowable roof thickness is significant to the bridge pile foundation. The underlying cave leads to the decrease of the bearing capacity of the bridge pile foundation. It is of great significance to ensure the safety of bridge foundation to consider the size of cave and the strength of roof of cave in the design.
2. The increasing cave height has little effect on the bridge pile foundation. The vertical bearing characteristics of the piles are greatly affected by the increasing cave span and roof thickness. When the span is greater than 9 m × 9 m or the roof is thinner than 3.0 m, the bearing capacity’s decrease of the bridge pile foundation must be considered cautiously.
3. The axial force of the pile decreases more faster with the increasing cave span and decreasing roof thickness. It is because that more forces are transferred to surrounding soils by rubbing. At this time, the strength of cave roof is very important to ensure the stability of pile foundation.
4. The tip resistance ratio of the pile on a cave decreases firstly, then keep almost steady with the increasing span. The side resistance ratio keeps steady, when the span exceeds 9 m × 9 m. The tip resistance ratio of the pile on a cave gradually increases with the increasing roof thickness.

## Data Availability

The data used to support the findings of this study are included within the article.
